# Supplementary material for: A fault-intrusion-tolerant system and deadline-aware algorithm for scheduling scientific workflow in the cloud
Source: PeerJ Comput Sci. 2021 Nov 2;7:e747. doi: 10.7717/peerj-cs.747 (PMC8576554; doi:10.7717/peerj-cs.747)
Supplement: Supplemental Information 6 [file peerj-cs-07-747-s006.zip › Myproject1.1/docs/org/cloudbus/cloudsim/provisioners/PeProvisioner.html]

PeProvisioner


JavaScript is disabled on your browser.


- Overview
- Package
- Class
- Use
- Tree
- Deprecated
- Index
- Help

- Prev Class
- Next Class

- Frames
- No Frames

- All Classes

- Summary:
- Nested |
- Field |
- Constr |
- Method

- Detail:
- Field |
- Constr |
- Method


org.cloudbus.cloudsim.provisioners

## Class PeProvisioner

- java.lang.Object
- - org.cloudbus.cloudsim.provisioners.PeProvisioner

- Direct Known Subclasses:
  :   PeProvisionerSimple

  ---

    

  ```
  public abstract class PeProvisioner
  extends java.lang.Object
  ```

  The Class PeProvisioner.

  Since:
  :   CloudSim Toolkit 2.0

- - ### Constructor Summary

    Constructors

    Constructor and Description

    `PeProvisioner(double mips)`

    Creates the new PeProvisioner.
  - ### Method Summary

    Methods

    | Modifier and Type | Method and Description |
    | --- | --- |
    | `abstract boolean` | `allocateMipsForVm(java.lang.String vmUid, double mips)` Allocates MIPS for a given VM. |
    | `abstract boolean` | `allocateMipsForVm(Vm vm, double mips)` Allocates MIPS for a given VM. |
    | `abstract boolean` | `allocateMipsForVm(Vm vm, java.util.List<java.lang.Double> mips)` Allocates MIPS for a given VM. |
    | `void` | `deallocateMipsForAllVms()` Releases MIPS used by all VMs. |
    | `abstract void` | `deallocateMipsForVm(Vm vm)` Releases MIPS used by a VM. |
    | `abstract java.util.List<java.lang.Double>` | `getAllocatedMipsForVm(Vm vm)` Gets allocated MIPS for a given VM. |
    | `abstract double` | `getAllocatedMipsForVmByVirtualPeId(Vm vm, int peId)` Gets allocated MIPS for a given VM for a given virtual Pe. |
    | `double` | `getAvailableMips()` Gets the available MIPS in the PE. |
    | `double` | `getMips()` Gets the MIPS. |
    | `double` | `getTotalAllocatedMips()` Gets the total allocated MIPS. |
    | `abstract double` | `getTotalAllocatedMipsForVm(Vm vm)` Gets total allocated MIPS for a given VM for all PEs. |
    | `double` | `getUtilization()` Gets the utilization of the Pe in percents. |
    | `protected void` | `setAvailableMips(double availableMips)` Sets the available MIPS. |
    | `void` | `setMips(double mips)` Sets the MIPS. |

    - ### Methods inherited from class java.lang.Object

      `clone, equals, finalize, getClass, hashCode, notify, notifyAll, toString, wait, wait, wait`

- - ### Constructor Detail


    - #### PeProvisioner

      ```
      public PeProvisioner(double mips)
      ```

      Creates the new PeProvisioner.

      Parameters:
      :   `mips` - overall amount of MIPS available in the Pe
  - ### Method Detail


    - #### allocateMipsForVm

      ```
      public abstract boolean allocateMipsForVm(Vm vm,
                              double mips)
      ```

      Allocates MIPS for a given VM.

      Parameters:
      :   `vm` - virtual machine for which the MIPS are being allocated
      :   `mips` - the mips

      Returns:
      :   $true if the MIPS could be allocated; $false otherwise


    - #### allocateMipsForVm

      ```
      public abstract boolean allocateMipsForVm(java.lang.String vmUid,
                              double mips)
      ```

      Allocates MIPS for a given VM.

      Parameters:
      :   `vmUid` - the vm uid
      :   `mips` - the mips

      Returns:
      :   $true if the MIPS could be allocated; $false otherwise


    - #### allocateMipsForVm

      ```
      public abstract boolean allocateMipsForVm(Vm vm,
                              java.util.List<java.lang.Double> mips)
      ```

      Allocates MIPS for a given VM.

      Parameters:
      :   `vm` - virtual machine for which the MIPS are being allocated
      :   `mips` - the mips for each virtual Pe

      Returns:
      :   $true if the MIPS could be allocated; $false otherwise


    - #### getAllocatedMipsForVm

      ```
      public abstract java.util.List<java.lang.Double> getAllocatedMipsForVm(Vm vm)
      ```

      Gets allocated MIPS for a given VM.

      Parameters:
      :   `vm` - virtual machine for which the MIPS are being allocated

      Returns:
      :   array of allocated MIPS


    - #### getTotalAllocatedMipsForVm

      ```
      public abstract double getTotalAllocatedMipsForVm(Vm vm)
      ```

      Gets total allocated MIPS for a given VM for all PEs.

      Parameters:
      :   `vm` - virtual machine for which the MIPS are being allocated

      Returns:
      :   total allocated MIPS


    - #### getAllocatedMipsForVmByVirtualPeId

      ```
      public abstract double getAllocatedMipsForVmByVirtualPeId(Vm vm,
                                              int peId)
      ```

      Gets allocated MIPS for a given VM for a given virtual Pe.

      Parameters:
      :   `vm` - virtual machine for which the MIPS are being allocated
      :   `peId` - the pe id

      Returns:
      :   allocated MIPS


    - #### deallocateMipsForVm

      ```
      public abstract void deallocateMipsForVm(Vm vm)
      ```

      Releases MIPS used by a VM.

      Parameters:
      :   `vm` - the vm


    - #### deallocateMipsForAllVms

      ```
      public void deallocateMipsForAllVms()
      ```

      Releases MIPS used by all VMs.


    - #### getMips

      ```
      public double getMips()
      ```

      Gets the MIPS.

      Returns:
      :   the MIPS


    - #### setMips

      ```
      public void setMips(double mips)
      ```

      Sets the MIPS.

      Parameters:
      :   `mips` - the MIPS to set


    - #### getAvailableMips

      ```
      public double getAvailableMips()
      ```

      Gets the available MIPS in the PE.

      Returns:
      :   available MIPS


    - #### setAvailableMips

      ```
      protected void setAvailableMips(double availableMips)
      ```

      Sets the available MIPS.

      Parameters:
      :   `availableMips` - the availableMips to set


    - #### getTotalAllocatedMips

      ```
      public double getTotalAllocatedMips()
      ```

      Gets the total allocated MIPS.

      Returns:
      :   the total allocated MIPS


    - #### getUtilization

      ```
      public double getUtilization()
      ```

      Gets the utilization of the Pe in percents.

      Returns:
      :   the utilization


- Overview
- Package
- Class
- Use
- Tree
- Deprecated
- Index
- Help

- Prev Class
- Next Class

- Frames
- No Frames

- All Classes

- Summary:
- Nested |
- Field |
- Constr |
- Method

- Detail:
- Field |
- Constr |
- Method
